# Navigating physician-scientist careers: insights from American Board of Internal Medicine Research Pathway graduates

**DOI:** 10.1172/jci.insight.195683

**Published:** 2026-08-24

**Authors:** Emily J. Gallagher, Barbara I. Kazmierczak, Michael Kisielewski, Nathan Schoettler, Clifford J. Steer, Robert A. Baiocchi, Jatin M. Vyas, Kyu Y. Rhee, Patrick J. Hu, Carlos M. Isales, Paul R. Conlin, Christopher D. Kontos, Michael Melfe, Furman S. McDonald, Don C. Rockey, Christopher S. Williams

**Affiliations:** 1 Division of Endocrinology, Diabetes and Bone Disease, Department of Medicine, Icahn School of Medicine at Mount Sinai, New York, New York, USA.; 2 Department of Microbial Pathogenesis, Department of Medicine (Infectious Diseases), Yale University School of Medicine, New Haven, Connecticut, USA.; 3 Alliance for Academic Internal Medicine, Alexandria, Virginia, USA.; 4 Section of Pulmonary and Critical Care Medicine, Department of Medicine, University of Chicago, Chicago, Illinois, USA.; 5 Department of Medicine, University of Minnesota Medical School, Minneapolis, Minnesota, USA.; 6 Division of Hematology, Department of Internal Medicine, Comprehensive Cancer Center, The Ohio State University, Columbus, Ohio, USA.; 7 Division of Infectious Disease, Department of Medicine, Columbia University, Vagelos College of Physicians and Surgeons, New York, New York, USA.; 8 Division of Infectious Diseases, Department of Medicine, Weill Cornell Medicine, New York, New York, USA.; 9 Departments of Medicine and Cell and Developmental Biology, Vanderbilt University Medical Center, Nashville, Tennessee, USA.; 10 Departments of Medicine, Neuroscience and Regenerative Medicine, Medical College of Georgia at Augusta University, Augusta, Georgia, USA.; 11 VA Boston Healthcare System and Harvard Medical School, Boston, Massachusetts, USA.; 12 Department of Medicine, Duke University Medical Center, Durham, North Carolina, USA.; 13 American Board of Internal Medicine, Philadelphia, Pennsylvania, USA.; 14 J. Edwin Wood Clinic of Pennsylvania Hospital, Philadelphia, Pennsylvania, USA.; 15 Division of Gastroenterology and Hepatology and Digestive Disease Research Center, Medical University of South Carolina, Charleston, South Carolina, USA.; 16 Department of Medicine, Division of Gastroenterology, Vanderbilt University Medical Center, Nashville, Tennessee, USA.; 17 Veterans Affairs Tennessee Valley Health Care System, Nashville, Tennessee, USA.; 18 Vanderbilt Ingram Cancer Center, Nashville, Tennessee, USA.

## Abstract

The American Board of Internal Medicine Research Pathway (ABIM RP) is designed to train physician-scientists by shortening postgraduate clinical training and increasing research time. We surveyed ABIM RP graduates to understand career trajectories, research engagement, and barriers to physician-scientist retention in academic research careers. We performed an anonymous, structured, web-based survey of 700 ABIM RP current and former trainees (2011–2022). We received 105 survey responses (97 RP graduates). The survey queried about demographics, training background, career outcomes, research involvement, and perceived barriers to retention in physician-scientist careers. Responses were stratified by trainee status, gender, and Medical Scientist Training Program (MSTP) participation, and select results were compared to test for statistical associations. Most respondents (79%) remained in academia, with 61% engaged in research-dominant roles. More MSTP graduates held research-focused academic positions than non-MSTP graduates. The most commonly reported barriers to sustaining research careers were funding instability, financial concerns, and lack of protected research time. Although many of the trainees in the ABIM RP who completed the survey remained in academia, systemic barriers such as funding instability and gaps in institutional support persist. Strategies to enhance financial stability, protected research time, and mentorship appear to be necessary to enhance physician-scientist career retention.

## Abstract 2

The study reports a survey of ABIM Research Pathway graduates to understand their research engagement, and barriers to physician-scientist retention in academic research careers.

## Introduction

Thirty years ago, the American Board of Internal Medicine (ABIM) formalized a residency curriculum, known as the Research Pathway, designed to train physician-scientists by integrating clinical training with research experience. The pathway provides a formal track culminating in ABIM board certification with two years of clinical internal medicine residency training and requires three years of research for general internists. Subspecialists extend the training to 4–5 years after residency. The total time in postgraduate training in the Research Pathway is therefore five to 7 years, depending on subspeciality (1–3). The purpose of the Research Pathway was to (a) shorten the time in clinical training for those committed to physician-scientist careers; (b) provide a structured training program to develop clinical and research skills; and (c) maintain a strong physician-scientist presence in the internal medicine workforce.

In 2012, the ABIM published outcomes of 1,009 individuals who matriculated in the Research Pathway and took the internal medicine certification exam for the first time between 1993 and 2008. They found that 22% of Research Pathway trainees were women, the mean age at initial internal medicine exam was 34 years, and first-attempt pass rates were slightly higher than those of categorical training program graduates. Of 166 Research Pathway graduates who subsequently enrolled in Maintenance of Certification and completed a Practice Characteristics Survey, 63% were academic faculty, 10% were not clinically active, and 27% were in a private group practice. Physicians who completed the research pathway devoted, on average, 37% of their professional time to medical research (1). Also in 2012, the Alliance for Academic Internal Medicine (AAIM) conducted a survey of Research Pathway graduates from 1995 to 2007. In that survey, 65% of graduates had a PhD prior to starting the Research Pathway, 72% held academic faculty positions, 9% were in industry, 11% were in private practice, and others were in nonmilitary government roles, or nonmedical, nonresearch careers. Respondents reported that they spent an average of 59% of their time pursuing research (2). Thus, during the early period after the launch of the ABIM Research Pathway, most graduates who responded to practice surveys remained in academic faculty positions.

Maintaining the physician-scientist workforce is essential for advancing medical research and translating scientific discoveries into clinical practice (4). We wished to explore the career outcomes, research engagement, perceptions about physician-scientist careers, and challenges faced by recent ABIM Research Pathway graduates. We took into account the degree type, as a previous study of US MD-PhD program matriculants from 1993 to 2000 reported that more graduates of long-standing Medical Scientist Training Program–funded (MSTP-funded) programs reported career preference as full-time faculty research scientists (62%), compared with graduates of non-MSTP-funded programs (32%) (5). Additionally, in studies of Canadian MD-PhD graduates, researchers found that physicians who obtained a PhD after medical school had more research focused careers than those who obtained a PhD prior to medical school, and completing a master’s degree prior to MD-PhD training was a negative predictor of sustained research involvement (6, 7). Understanding these factors is crucial for identifying systemic barriers and informing policies to enhance physician-scientist retention.

To address these knowledge gaps, we conducted a survey of current and former ABIM Research Pathway trainees to evaluate their career pathways, research involvement, and perceived challenges. The objectives of this study were to (a) characterize the demographics and training backgrounds of recent ABIM Research Pathway participants; (b) assess their career outcomes, including workplace setting and research engagement; (c) evaluate their confidence in sustaining a physician-scientist career; and (d) identify perceived barriers contributing to attrition from academic medicine. By analyzing these domains, this study aimed to provide insights into the evolving landscape of the physician-scientist workforce and inform strategies to enhance support for trainees and early-career physician-scientists.

## Results

### Cohort demographics.

There were 105 survey respondents, which included 8 (8%) current internal medicine residents or fellows in training and 97 (92%) graduates from the ABIM Research Pathway. Due to the small number of current trainees, the results presented here include only the 97 graduates of the ABIM Research Pathway. Thirty-four respondents (34%) self-identified as women, 59 (61%) were self-identified men, and 4 (4%) preferred not to state their gender. Survey respondents could select one or more race and ethnicity categories; therefore, percentages are not mutually exclusive and do not sum to 100%. A majority of respondents self-identified as White (n = 64, 66%), followed by East Asian (n = 10, 10%), Hispanic, Latino, or of Spanish origin (n = 8, 8%), and South Asian (n = 8, 8%). Other race and ethnicity categories represented in the survey included American Indian or Alaska Native, Black or African American, Southeast Asian, and Middle Eastern or North African. Eight respondents (8%) preferred not to answer. The average age at completion of Research Pathway residency training for all survey respondents was 33 (±3 SD) years.

### Training prior to starting the Research Pathway.

**Figure 1:**
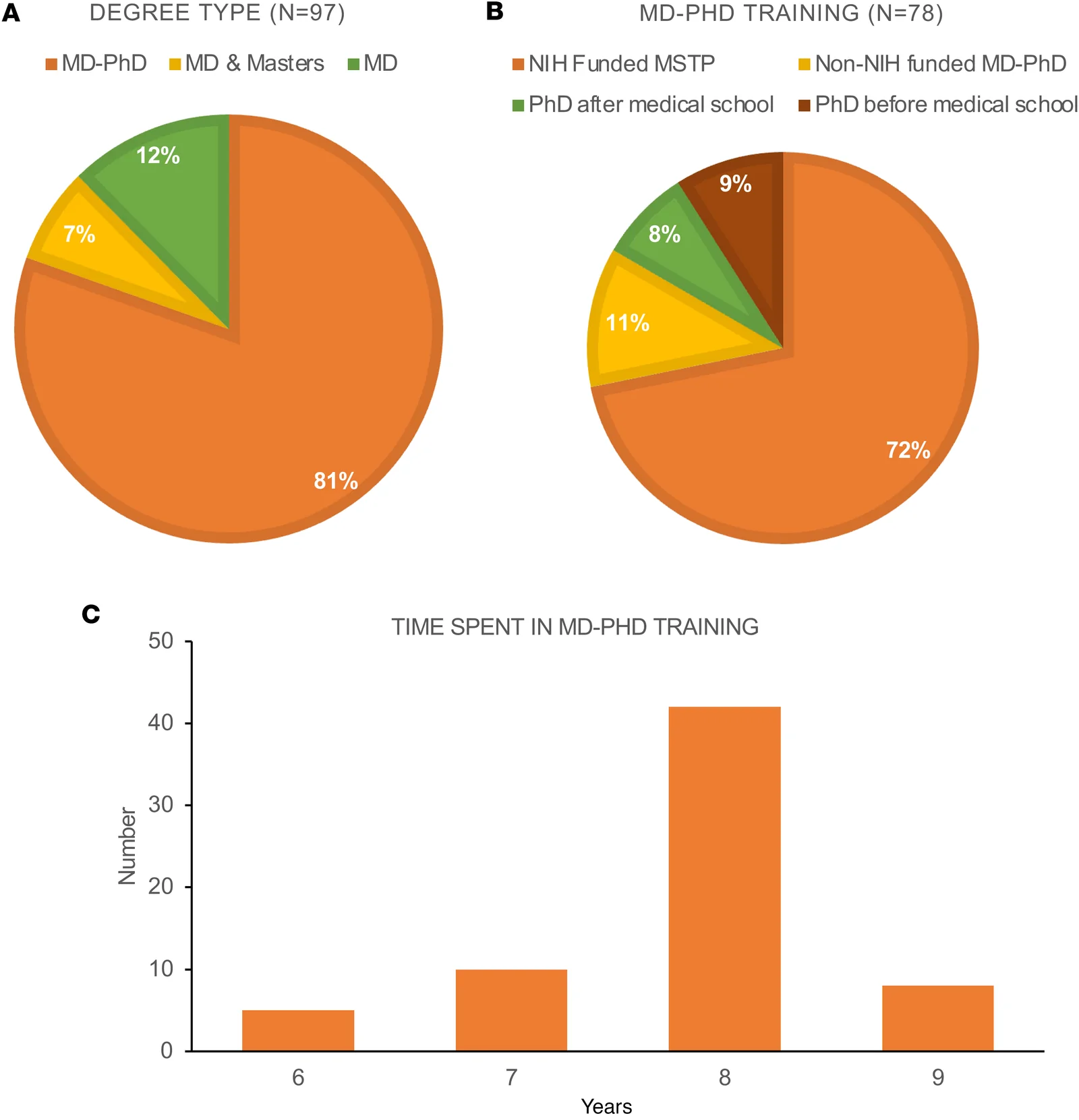
Degree types and MD–PhD training characteristics of survey respondents. (**A**) Degree types for all survey respondents (*n* = 97). (**B**) Type of training for survey respondents who reported having MD and PhD degrees (*n* = 78). (**C**) Number of years in MD-PhD training for those who completed combined MD-PhD degrees.

The majority of survey respondents (*n* = 78/97, 80%) reported having an MD-PhD (or international equivalent degree); 12 respondents with MD-PhDs also reported having Master’s degrees (Figure 1A). Of those with PhD degrees (Figure 1B), 56 of 78 (72%) obtained their MD-PhD through an NIH-sponsored MSTP, 9 (13%) received their MD-PhD through a non-NIH funded program, 7 (10%) received their PhD prior to medical school matriculation, and 6 (8%) received their PhD after graduating from medical school. Twelve respondents (12%) had MD degrees without an additional degree, and 7 (7%) had an MD with Master’s degrees (MA, MBA, MS, MPH) (Figure 1A). None of the respondents held DO degrees. For those who completed combined MD-PhD training in MSTP or non-NIH funded dual-degree programs (*n* = 64), the average time spent in undergraduate medical school training was 8 (±0.7 SD) years (Figure 1C).

### Subspecialty training of Research Pathway graduates.

**Figure 2:**
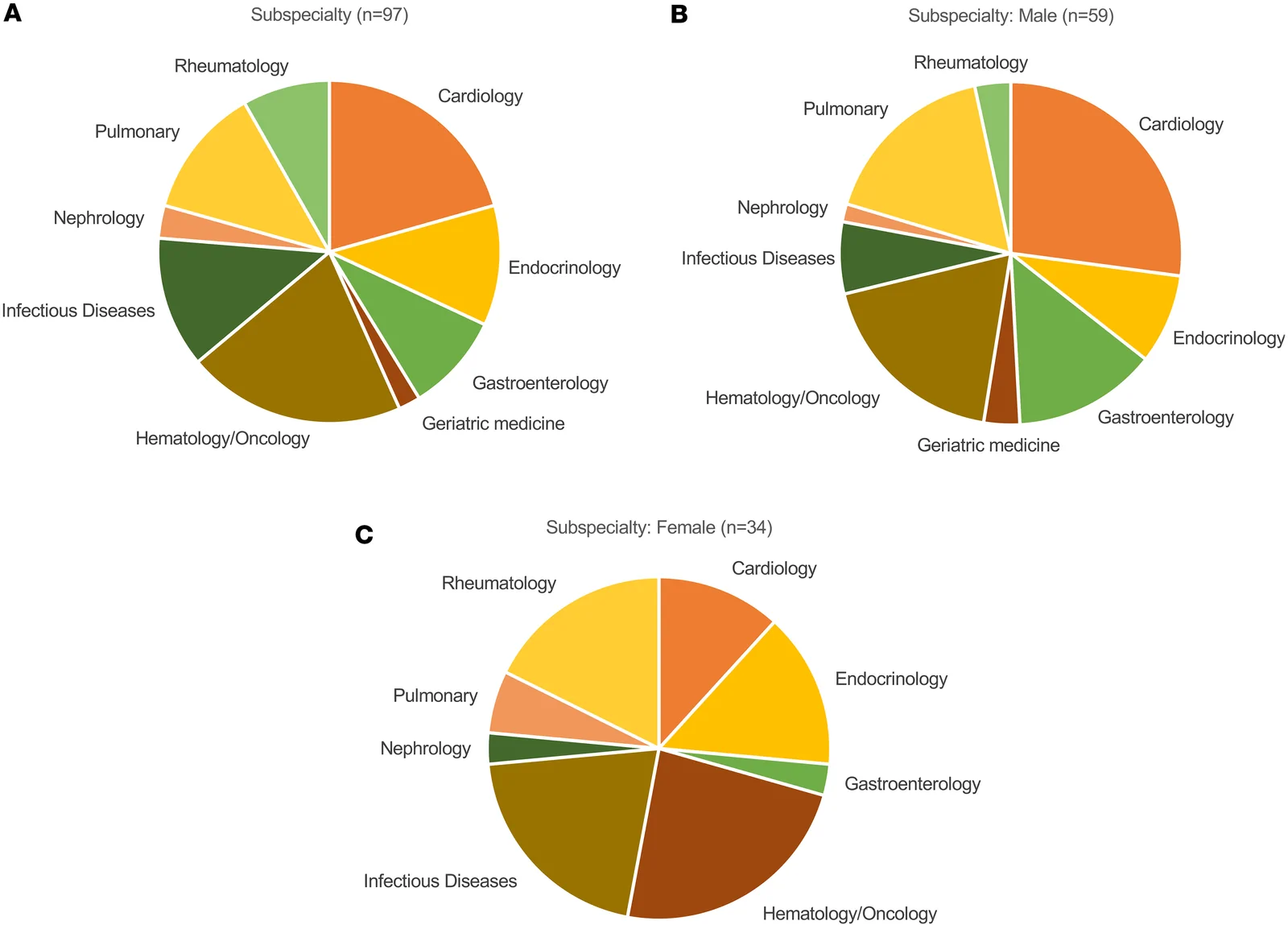
Subspecialty training for all graduates of the American Board of Internal Medicine Research Pathway. (**A**) Subspecialty training among all graduates (*n* = 97).(**B**) Subspecialty training among male graduates. (**C**) Subspecialty training among female graduates.

All of the 97 individuals who completed postgraduate Research Pathway training reported pursuing subspecialty training. Of those who graduated from subspecialty training programs, the most commonly reported subspecialties were cardiology (*n* = 20/97, 21%) and hematology/oncology (*n* = 20, 21%), with the least common being nephrology (*n* = 3, 3%) and geriatric medicine (*n* = 2, 2%) (Figure 2A). The most common field of subspecialty training for men was cardiology (*n* = 16/59 men, 27%) and that for women was hematology/oncology (*n* = 8/34 women, 24%), with a statistically significant difference in subspecialty distribution between men and women (*P* = 0.022) (Figure 2, B and C). There were no statistically significant differences in subspecialty choices between MSTP graduates and non-MSTP graduates.

### Professional activities of Research Pathway graduates.

**Figure 3:**
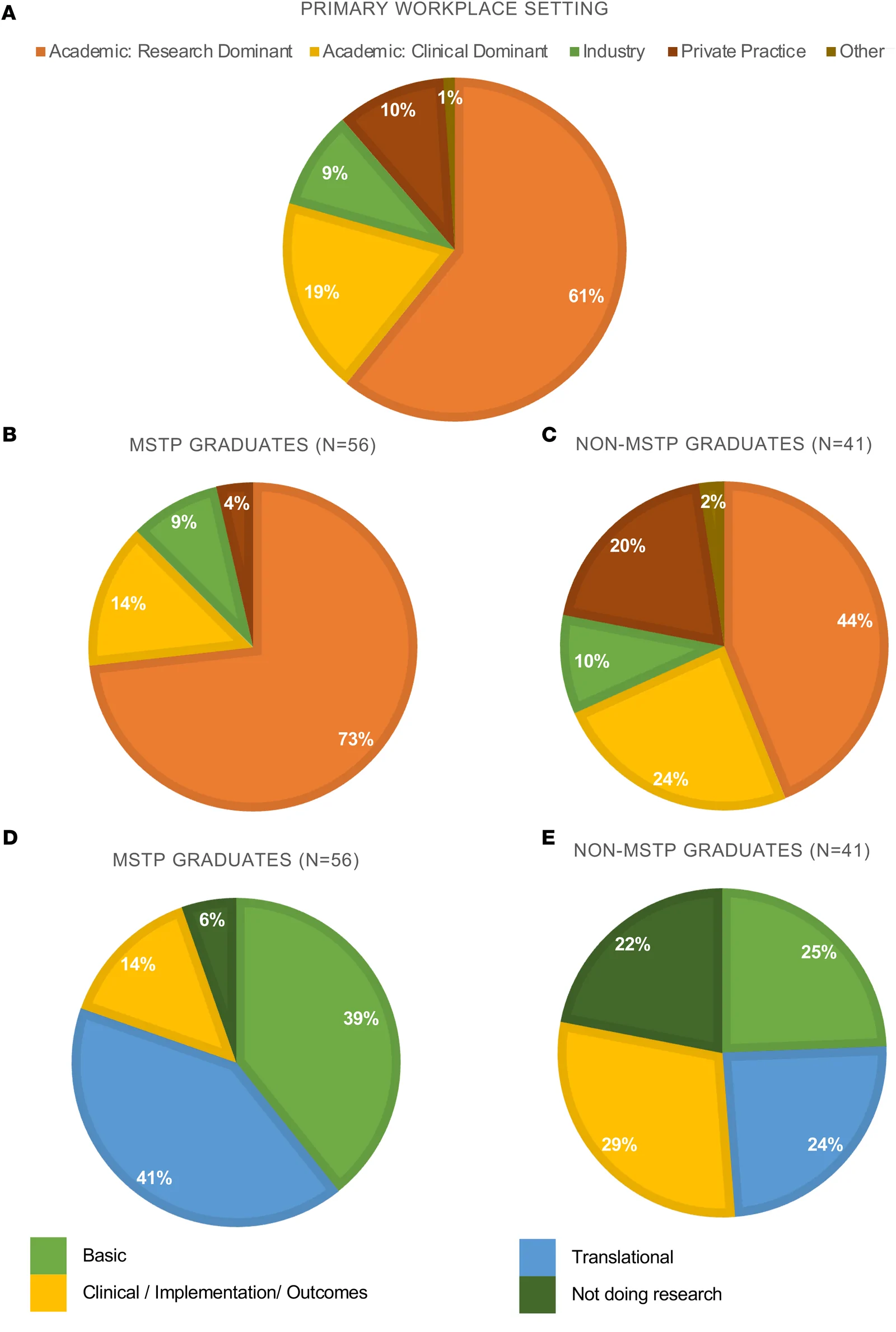
Workplace settings and research activities of ABIM Research Pathway graduates. (**A**) Distribution of primary workplace setting for survey respondents who were graduates of the ABIM Research Pathway (*n* = 97) and for (**B**) graduates of MSTP and (**C**) non-MSTP (**C**) programs. Reported type of research being performed by (**D**) MSTP and (**E**) non-MSTP graduates of the ABIM Research Pathway.

The median year of completing all postgraduate training was 2017 (IQR, 6 years); and, therefore, a median of 7 years had passed between training and completion of the survey. Seventy-seven of the 97 graduates (79%) reported that their primary workplace setting was in academia, with 59 of 97 (61%) reporting academic appointments with 50% or more of their time spent in research (research-dominant); and 18 (19%) reporting academic appointments with more than 50% clinical activities (clinical-dominant). Among those not in an academic setting, 9 (9%) reported their primary workplace as industry, and 10 (10%) reported their primary workplace being in private practice (Figure 3A). There were no associations in the distribution of primary workplace between those who had graduated prior to 2017 and those who graduated in or after 2017 or between men and women. However, MSTP graduates reported research-dominant positions in academia to a greater extent (41/56, 73%) than non-MSTP graduates (18/41, 44%, *P* = 0.012, Figure 3, B and C). With respect to what best described their primary research activity, 55 of 97 (67%) of the Research Pathway graduates reported pursuing basic or translational research; 20 (19%) described working in clinical, implementation, or outcomes research; and 12 (12%) reported no current research activities. Of MSTP graduates, 45 of 56 (80%) reported doing basic or translational research, whereas 20 of 41 (48%) non-MSTP graduates reported doing basic or translational research. Fewer MSTP graduates (8/56, 14%) were involved in clinical, implementation, or outcomes research than non-MSTP graduates (12/41, 28%), and fewer MSTP graduates reported not currently doing research (3/56, 5%) than non-MSTP graduates (9/41, 22%, *P* = 0.010; Figure 3, D and E).

### Confidence of succeeding in a career as a physician-scientist.

**Figure 4:**
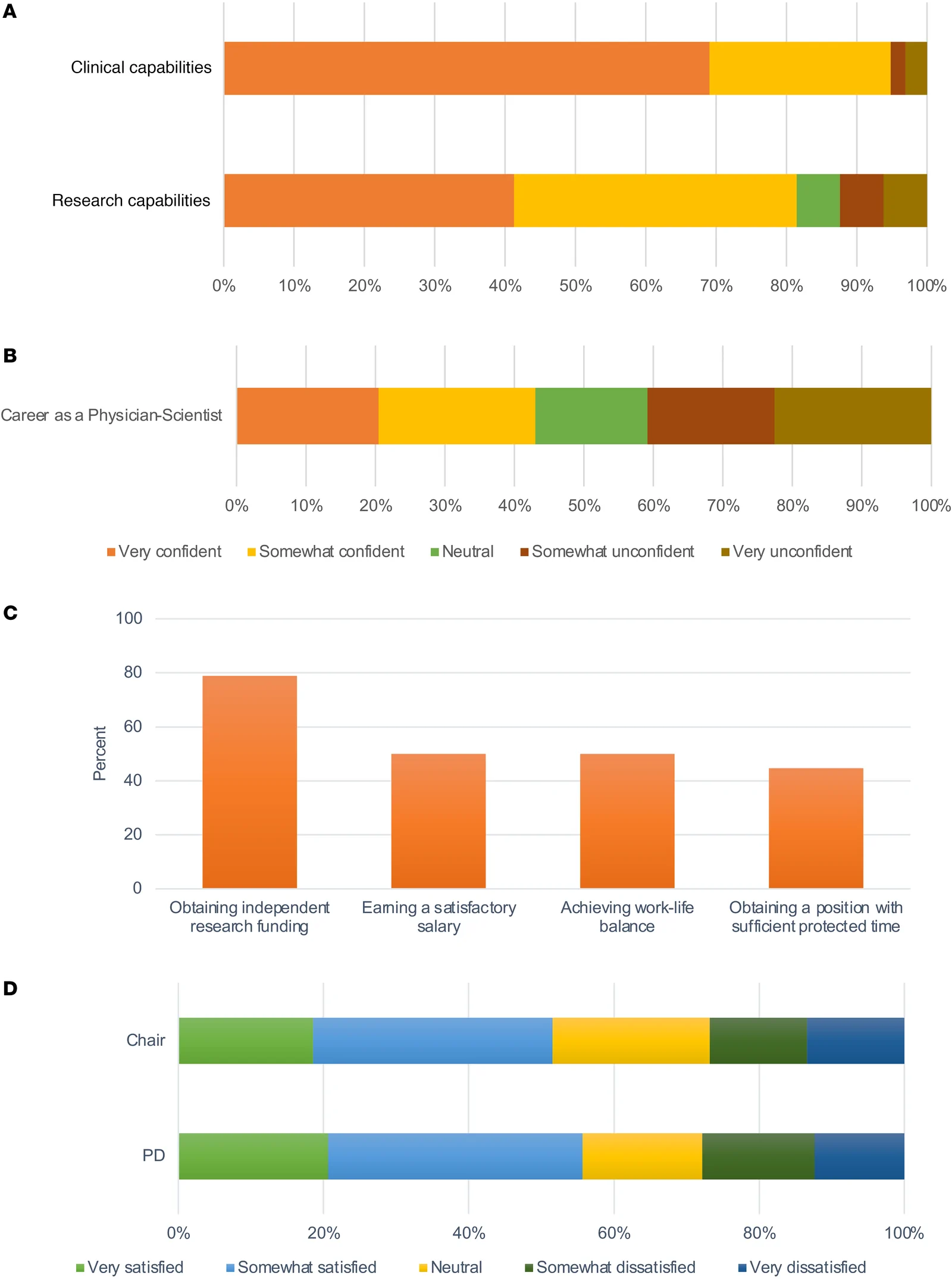
Confidence in physician-scientist careers and perceived institutional support among ABIM Research Pathway graduates. Distribution of responses to Likert scale questions asking ABIM Research Pathway graduates (*n* = 97) (**A**) about their confidence in their clinical and research capabilities and (**B**) ability to succeed in a physician-scientist career. (**C**) The concerns reported by those that said they were somewhat or very unconfident in their ability to succeed in a career as a physician-scientist. (**D**) Levels of satisfaction or dissatisfaction that survey respondents (*n* = 105) had with their program director (PD) or chair’s understanding of the challenges faced by trainees in the American Board of Internal Medicine Research Pathway.

When asked how confident graduates were in their research capabilities, 79 of 97 (81%) reported being somewhat or very confident. When asked about their confidence in their clinical abilities, 92 of 97 (95%) reported being somewhat or very confident (Figure 4A). However, when asked about their confidence in their ability to succeed in careers as physician-scientists, almost equal percentages were somewhat or very confident 40 of 97 (41%) as were somewhat or very unconfident 38 of 97 (39%) (Figure 4B). There was no statistically significant difference in confidence between those who graduated prior to 2017 and those who graduated in or after 2017. We did not specifically ask about K or R grants obtained or academic rank. Based on reported year of completion of training, 14 (14%) were 2–3 years after training and were likely instructors or early assistant professors; 28 (29%) were 4–6 years after training and were likely early-to-mid assistant professors; 20 (21%) were 7–9 years after training and were likely assistant professors or associate professors; and 35 (36%) were 10–13 years after training and were likely at the associate professor rank. These academic rank estimates are based on the Prospective Study of Promotion in Academia (8).

Of those who reported being somewhat or very unconfident, the commonly reported concerns were being able to obtain independent research funding (*n* = 30/38, 79%), followed by having a satisfactory salary (*n* = 19/38, 50%), achieving a work-life balance (*n* = 19, 50%), and having sufficient protected research time (*n* = 17, 45%, Figure 4C). Of those who responded “Other,” the comments included finding satisfaction in an alternative career path and lack of institutional support.

When asked how satisfied they were with their program director’s understanding of the challenges faced by residents and fellows in the Research Pathway, 61 of 105 (58%) reported being somewhat or very satisfied. When the same question was asked in relation to their department chair, 56 of 104 (54%) reported being somewhat or very satisfied (Figure 4D). When asked how satisfied they were with pursuing a research career similar to their mentors, 58 of 105 (55%) reported being somewhat or very satisfied.

### Perceptions on why individuals leave the academic physician-scientist career path.

In response to a question that asked what they believed were the greatest reasons for individuals to leave physician-scientist career paths, 78 of the 97 graduates (80%) entered free-text responses. The commonly reported reasons for individuals to leave the physician-scientist career path were (a) lack of research funding/instability of research funding (*n* = 43/78, 55%); (b) lack of financial stability/better paid alternative careers (*n* = 39/78, 50%); (c) increased clinical or administrative responsibilities and lack of protected research time (*n* = 25/78, 32%); (d) burnout, stress, feeling undervalued, and finding the career unfulfilling (*n* = 24/78, 31%); and (e) lack of institutional support (*n* = 24/78, 31%). Other reasons cited less frequently included caregiver/family responsibilities (*n* = 13, 17%), long duration of training (*n* = 12, 15%), lack of career advancement *n* = 7, 9%), work/life balance (*n* = 8, 10%), poor mentorship (*n* = 4, 5%), interest in something other than research (*n* = 4, 5%), and unhealthy work culture/environment (*n* = 3, 4%). A majority of those in research-dominant careers (*n* = 33/49, 67%) thought lack of research funding was a common reason why individuals leave research careers, and, of those in private practice, 5 of 7 (71%) believed compensation and financial stability were common causes. For those in clinical-dominant academic roles, compensation, financial stability (*n* = 6/13, 46%), and increased clinical or administrative responsibilities/lack of protected research time (*n* = 6/13, 46%) were the most common reasons. Among survey respondents in industry, 3 of 8 (38%) cited the duration of training, financial stability/compensation, and protected time as the most common reasons people leave the physician-scientist career path.

## Discussion

Our findings highlight key insights into the career trajectories, research engagement, and challenges faced by ABIM Research Pathway graduates. A relatively high proportion of Research Pathway graduates who responded to the survey remain in academia, with a majority engaged in research-dominant roles. This underscores the success of the pathway in fulfilling its mission of training physician-scientists. However, we also found differences in long-term career sustainability among non-MSTP trainees, who were significantly less likely to hold research-intensive academic positions.

Concerns about funding instability appear to reflect a pervasive and ongoing challenge for physician-scientists, with more than a third of graduates from the Research Pathway very or somewhat unconfident in their ability to have a secure and stable funding situation. Funding insecurity not only discourages early-career researchers from remaining in academia but is also associated with burnout and career dissatisfaction (9). Furthermore, concerns about earning a satisfactory salary, work-life balance, and protected research time are themes consistent with prior studies (10, 11). These themes were echoed in responses to the question regarding why Research Pathway graduates leave physician-scientist career paths. These findings suggest that protected research time, fair and guaranteed salaries, and sustainable funding models need to be considered to reduce attrition of physician-scientists.

There was a higher proportion of women in our survey respondents (33% vs. 22%) when compared with the ABIM survey conducted in 2012 (1). While the distribution of subspecialty training differed between men and women, there was no statistically significant difference between their professional settings. The proportion of underrepresented minorities in our survey respondents was very low, although it was similar to the percentage of underrepresented matriculants to all MD-PhD programs between 2009 and 2018 (12). Previous studies have reported persistently higher attrition rates for Black students than their peers from MD-PhD programs (13). The number of respondents in our survey who self-identified as underrepresented minorities was too small to meaningfully compare responses between groups. Prior studies have reported that fewer women and underrepresented minorities have NIH grants, suggesting that there may be greater attrition during the postgraduate training phase (14).

Given the increasing number of gap years trainees take before entering MSTP programs (15), it was surprising that the average age at initial internal medicine exam was 33.9 (±3.4 SD) years in the 2012 survey, and the average age at completion of Research Pathway training in our survey was 33 (±3 SD) years. As Research Pathway trainees are ineligible to take the initial internal medicine certification exam until 1 year after completing residency training, the age of the graduates responding to our survey should be 1 year older when taking their initial certification exam. Therefore, the age of entry into the initial part of the training remained similar to that in the 2012 study. In the study by Brass and colleagues evaluating the increasing gap years between undergraduate and MSTP matriculation, the cohort included those matriculating between 2013 and 2020 (15). Individuals in our survey reported spending on average 8 years in MD-PhD training, and the graduates of the ABIM Research Pathway spend a minimum of 5 additional years in postgraduate training; therefore, almost all those who responded to our survey would have matriculated into medical school before 2013. Therefore, the effects of the increasing number of gap years before MSTP matriculation are likely yet to be reflected in the age of those graduating from the ABIM Research Pathway.

In the 2012 AAIM survey, 89% reported having a PhD prior to starting residency, compared with 79% of our survey respondents (2). While 72% of respondents in the 2012 survey held faculty positions, 79% of the graduates in our survey had positions in academia. There were similar proportions of Research Pathway graduates in industry and private practice in the two surveys. The significant association between MSTP participation and research-dominant career paths may suggest that structured support during medical school plays a pivotal role in fostering long-term research engagement. These findings are consistent with prior studies examining overall retention in research careers from US and international MD-PhD programs (16, 17). The study was not designed to examine if there was more retention in academic research careers for MSTP graduates who participated in the ABIM Research Pathway compared with those who completed categorical residency training, but whether the Research Pathway is providing benefits to these graduates is an important topic for a future study to assess. We did not ask about educational debt, which has previously been reported to be an independent predictor of attrition from physician-scientist careers in US and Canadian studies and may be an unaccounted for confounder in this study (5, 6). We also did not ask about medical school ranking or geographical region, so also do not know if these factors affected experiences. Even though the ABIM Research Pathway provides a training framework, postgraduate physician-scientist training programs often lack the structure of an MSTP (18, 19). Targeted intervention to provide more structure to postgraduate training programs, particularly for non-MSTP trainees, may help to retain individuals in physician-scientist careers. One such structured postgraduate training program is the NIH R38 Stimulating Access to Research in Residency (StARR) program that typically provides research support, career development, and mentorship to non-MSTP graduates during residency training (20). While academic institutions can design the StARR training program to best meet the needs of their trainees, specialty board (e.g., ABIM) approval is required to ensure participating trainees meet the eligibility requirements for initial ABIM certification exam. The R38 programs were first funded in 2018, so it is too early to determine the long-term outcomes of their trainees at this point in time.

### Limitations.

This study provides critical insights into the career paths and challenges of ABIM Research Pathway graduates but has several limitations that should be considered. The most important limitation of this study is that the low response rate introduces the possibility of nonresponse bias and may limit the generalizability of our findings. Individuals who remained in academic medicine or research-focused careers may have been more likely to respond than those who left these career paths. Consistent with this possibility, a previous survey of MD-PhD graduates found lower academic retention among nonresponders than responders (16). Therefore, the true rates of academic retention and research engagement reported in this study may overestimate the true rates among all ABIM Research Pathway graduates. It is important to consider this limitation when comparing our results with those of prior studies or extrapolating them to the wider physician-scientist population. Despite this limitation, the survey provides important insights into the experiences and challenges perceived by ABIM Research Pathway graduates regarding funding instability, compensation, protected research time, and institutional support, all of which were reported by the respondents who largely remained engaged in physician-scientist careers. However, we did not assess whether these factors caused individuals to leave the physician-scientist career pathway. We speculate that all of these factors come into play at some level in all physician-scientists but that specific reasons that individuals leave the pathway are highly individual.

Our findings are limited to graduates of the ABIM Research Pathway and may not be generalizable to physician-scientists in other specialties. While concerns regarding funding insecurity, work-life balance, and insufficient institutional support may be common across disciplines (9), the relative importance of these factors may differ substantially by specialty. Surgical scientists often contend with intense clinical productivity demands and limited time for research, and surgical departments have historically had lower rates of NIH funding than many medical specialties (21–23). In pediatrics, the physician-scientist workforce has faced a steep decline over the past 40 years, which may reflect economic disincentives and challenges in recruiting and retaining research-focused faculty (24, 25). Family medicine has long faced underinvestment in research training infrastructure and has among the lowest rates of research engagement among MD-PhD graduates (16, 26), whereas pathology, despite ongoing recruitment challenges, has among the highest rates of sustained research engagement for MD-PhD graduates who enter the field (16, 27). These observations suggest that the challenges identified in the present study likely reflect a combination of broadly shared barriers as well as specialty-specific barriers. Additional studies using comparable methods across physician-scientist training pathways in other specialties would help determine which challenges are universal and which require specialty-specific interventions. Until such data are available, extrapolating our findings from ABIM Research Pathway graduates to physician-scientist populations in other specialties should be done cautiously.

Despite these limitations, the findings contribute to the ongoing discussion about sustaining the physician-scientist workforce and provide some actionable insights for institutions and policymakers aiming to improve career retention among physician-scientists. Coordinated specialty-specific data collection and synthesis is needed to understand unique challenges and opportunities, to guide tailored interventions, and, ultimately, to sustain the physician-scientist workforce.

### Conclusions.

Our findings highlight the successes and challenges of the ABIM Research Pathway in sustaining physician-scientist careers. A majority of graduates who responded to the survey remain in academia in research-dominant roles, but funding instability, financial anxieties, and lack of protected time remain major concerns. These are almost certainly barriers to their long-term retention in research careers. We conclude that addressing these structural issues, both at institutional and national levels, will be essential to maintaining physician-scientists in the internal medicine workforce.

## Methods

The survey was web-based and developed by the AAIM Research Committee and the AAIM Surveys and Research professional staff in collaboration with the ABIM. The survey instrument was developed through an iterative process by a subset of the AAIM Research Committee, in collaboration with the ABIM. AAIM Surveys and Research professional staff provided input on best practices for survey design. To improve upon the content validity, the web-based survey was pilot-tested by the complete Research Committee (15 members) and by 6 internal medicine fellows in physician-scientist training programs. All pilot test comments were aggregated by Surveys staff, reviewed and addressed by the authors, and the survey instrument was revised accordingly. The survey was fielded in Qualtrics Surveys (version XM). It consisted of 52 multiple choice, Likert scale, and free-text questions and included branching or conditional logic for select questions (i.e., not all respondents were presented with every question). The questions pertained to demographics, qualifications and experiences prior to entering the Research Pathway, subspecialty training, practice settings of graduates, and their perceptions of challenges facing physician-scientists. The names and contact information of ABIM Research Pathway trainees and graduates were not available to the AAIM Research Committee or AAIM Surveys staff; rather, ABIM distributed the survey URL anonymously by email, on behalf of AAIM, to 713 current (as of June 2024) or former (from 2011 to 2022) trainees in the Research Pathway. ABIM also distributed three email reminders between June and August 2024, and AAIM sent a general reminder to the entire membership via the electronic/email newsletter *AAIM Connection*, requesting recipients to ask their current or former trainees in the ABIM Research Pathway if they received the email invitation.

To ensure participant anonymity, no identifiable population-level data (e.g., stratifying characteristics) were provided to AAIM in advance of the survey to describe the population, such as the differential between current trainees and graduates. Similarly, no data about status (e.g., resident or fellow) or primary workplace setting for graduates were obtained in advance. After reducing the final population size to an estimated 700 (to account for undeliverable rates), the unadjusted response rate was 15%. The complete responses were stratified by aggregating “resident or fellow” into one group and “graduates” into another. Age at completion of residency training was calculated by subtracting year of birth from the year of completion of residency training. Any individual who reported spending 50% of their time in academia in research activities was classified as belonging to the “Academia: Research-dominant activities” group. Where more than one subspecialty was reported (e.g., pulmonary and critical care medicine or pulmonary and sleep medicine), they were grouped under one subspecialty (e.g., pulmonary). Qualitative free-text responses were analyzed using thematic analysis. Responses were first reviewed for familiarization and then coded into overarching themes and then reviewed.

### Statistics.

Data were analyzed using descriptive statistics. Categorical variables were summarized using frequencies and percentages, whereas continuous variables were reported as mean and standard deviations (±SD) or medians and IQR, as appropriate. χ^2^ tests were used to assess associations between categorical variables, such as subspecialty choice and gender or MSTP status. Fisher’s exact test was used when expected cell counts were below 5. Differences in continuous variables between groups were evaluated using 2-tailed *t* tests for normally distributed data and the Mann-Whitney *U* test for nonnormally/nonparametrically distributed data. Statistical significance was defined as *P* ≤ 0.05. All analyses were conducted using SPSS Statistics version 29.0.2.0 (IBM) and Stata SE version 18.0 (StataCorp).

### Study approval.

The study received an exempt determination by Pearl Institutional Review Board, Fishers, Indiana, USA (protocol 2024-0202-AAIM, IRB00007772), in accordance with the applicable federal regulations, according to 45 CFR 46.104(d) (2); written informed consent was therefore not required.

### Data availability.

Aggregate anonymous survey results will be made available from the corresponding author upon request. Supporting data values for each figure panel are provided in the Supporting Data Values file.

## Author contributions

RAB, PRC, EJG, PJH, CMI, BIK, MK, CDK, FSM, MM, KYR, DCR, NS, CJS, JMV, and CSW contributed to conceptualizing the study and the design of the survey instrument. MK and EJG analyzed the results of the survey, and RAB, PRC, EJG, PJH, CMI, BIK, MK, CDK, FSM, MM, KYR, DCR, NS, CJS, JMV, and CSW contributed to interpreting the data. EJG generated the initial manuscript draft that RAB, PRC, EJG, PJH, CMI, BIK, MK, CDK, FSM, MM, KYR, DCR, NS, CJS, JMV, and CSW contributed to revising and critically editing. EJG is responsible for the accuracy and integrity of the results.

## Conflict of interest

CDK received support from ICON Clinical Research LLC for events adjudication for clinical trials. CMI reports holding stock in Pfizer and having a patent application (6410508) filed for compounds targeting the aging process. DCR has received research support from the following: Akero, AstraZeneca, Durect, Galectin Therapeutics, Gilead Sciences, Intercept Pharmaceuticals, Ipsen, NovoNordisk, and Salix Pharmaceuticals. EJG reports consulting fees from Novartis. RAB reports advisory board income from Viracta and Atara as well as research support from CODIAK Biosciences and Prelude Therapeutics.

## Funding support

This work is the result of NIH funding, in whole or in part, and is subject to the NIH Public Access Policy. Through acceptance of this federal funding, the NIH has been given a right to make the work publicly available in PubMed Central.

- NIH grants T32GM136651 to BIK; T32 GM145449 and R01 HL156009 to CDK; T32 GM007347, R38 HL167237, and I01BX001426 to CSW; P30 DK123704, R25 DK137316 and P20 GM130457 to DCR; R38 HL172261, and R38 AI181012 to EJG; R38 AI174255 to KYR; R25 AI147393, R38 HL150212, and R38 AG070229 to JMV; and K08 HL153955 to NS
- Burroughs Wellcome Fund Physician-Scientist Institutional Award Program to KYR.

## Supplementary Material

Supporting data values

## Version 1 08/24/2026

Electronic publication
